# Predicting Breast Imaging-Reporting and Data System Classification of Palpable Breast Masses Using Ultrasound to Prioritize Mammography Queues

**DOI:** 10.14740/jocmr6409

**Published:** 2026-01-04

**Authors:** Sarisa Thinyu, Thanin Lokeskrawee, Takumi Sakata, Natthaphon Pruksathorn, Suppachai Lawanaskol, Jayanton Patumanond, Suwapim Chanlaor, Wanwisa Bumrungpagdee, Chawalit Lakdee

**Affiliations:** a Department of Radiology, Lampang Hospital, Lampang 52000, Thailand; b Department of Emergency Medicine, Lampang Hospital, Lampang 52000, Thailand; c Faculty of Medicine and Health Sciences, Yamaguchi University, 1-1-1 Minami-Kogushi, Ube, Yamaguchi 755-8505, Japan; d Chaiprakarn Hospital, Chiang Mai 50320, Thailand; e Clinical Epidemiology and Clinical Statistics Unit, Faculty of Medicine, Naresuan University, Phitsanulok 65000, Thailand; f Department of Radiology, Buddhachinaraj Phitsanulok Hospital, Phitsanulok 65000 Thailand

**Keywords:** Breast neoplasms, Mammography, Mammary ultrasonography, BI-RADS, Early detection of cancer

## Abstract

**Background:**

Breast cancer is the leading cause of cancer death in women worldwide. Breast imaging, usually mammography and/or ultrasound, is classified using the Breast Imaging-Reporting and Data System (BI-RADS). At Lampang Hospital, mammography delays of up to 5 months postpone diagnosis in 40% of breast cancer cases. An urgent queue for palpable breast masses was introduced, but nearly half were benign, leading to inefficient prioritization. This study aimed to develop a two-step model based on high-risk ultrasound features and compare it with reference BI-RADS classifications.

**Methods:**

This diagnostic prediction study collected retrospective data from Lampang Hospital between January 2021 and December 2023. Ultrasound images of 390 patients were independently reviewed by radiologists blinded to the reference BI-RADS classification. Stepwise multivariable risk difference regression analysis was applied to identify predictive characteristics from seven predefined ultrasound findings.

**Results:**

Three predictive characteristics were identified: shape, margin, and echo pattern. The two-step model showed excellent discrimination, with an area under the receiver operating characteristic curve (AuROC) of 0.9801 (95% CI, 0.9696–0.9907) in step 1 and 0.9623 (95% CI, 0.9411–0.9835) in step 2. Internal validation with 200 bootstrap cycles confirmed minimal optimism. Using prevalence-based cut points, the model achieved 88.5% accuracy, with 6.7% underestimation in BI-RADS 4–5 (predicted as 3) and overestimation not exceeding 3% in any category.

**Conclusions:**

A two-step ultrasound-based model using shape, margin, and echo pattern demonstrated excellent discrimination as well as high accuracy, with slightly increased underestimation and minimal overestimation. This re-scheduling strategy optimizes mammography queue prioritization, but external validation is required before clinical implementation.

## Introduction

Breast cancer was the most common cancer among women in 157 out of 185 countries in 2022, with 2.3 million new cases and 670,000 deaths reported globally [1, 2]. The objective of the World Health Organization (WHO) Global Breast Cancer Initiative (GBCI) is to reduce global breast cancer mortality by 2.5% per year. The three pillars for achieving this goal are health promotion for early detection, timely diagnosis, and comprehensive breast cancer management [1].

Mammography is widely accepted as the standard method for breast cancer screening and diagnosis, contributing to a reduction in patient mortality [3, 4]. Its diagnostic accuracy improves when combined with breast ultrasound [5, 6]. Radiologists report findings based on the American College of Radiology (ACR) Breast Imaging-Reporting and Data System (BI-RADS) fifth edition (2013) classification [7]. However, mammography can only be performed in hospitals equipped with both mammography machines and qualified radiologists.

At Lampang Hospital, a high volume of mammography referrals has led to prolonged waiting times—nearly 5 months—with almost 40% of breast cancer patients experiencing delayed diagnosis. To address this, the Department of Radiology implemented an expedited mammography queue system, guaranteeing appointments within 4 weeks for patients with clinically palpable breast lumps suspicious for malignancy. Nevertheless, nearly half of these urgent cases were ultimately diagnosed as simple cysts or benign masses, indicating that they were not truly urgent.

Ultrasound is a widely used, accessible, radiation-free imaging modality with high diagnostic sensitivity, capable of distinguishing between benign and suspicious breast masses [8–10]. If initial evaluation with breast ultrasound was utilized to assess palpable lumps beforehand, it could help stratify patients more appropriately and optimize mammography scheduling. This study aimed to develop a two-step model using high-risk ultrasound features of palpable breast masses and to compare its performance with BI-RADS classifications from reference imaging.

## Materials and Methods

### Study design

This diagnostic prediction study used a retrospective cross-sectional design conducted in the Radiology Department of Lampang Hospital, a tertiary referral center in northern Thailand with an annual volume of approximately 240,000 radiology visits. The study period spanned January 2021 to December 2023. Eligible participants were women aged ≥ 18 years who presented with palpable breast masses and underwent mammography and/or breast ultrasound as part of their diagnostic evaluation. In routine clinical practice, final BI-RADS classifications are assigned by board-certified radiologists with more than 5 years of experience based on mammography and ultrasound together or on ultrasound alone when mammography is insufficient, such as in women with dense breast tissue. Data collected included patient age, ultrasound features assessed according to the ACR BI-RADS fifth edition, and the corresponding reference BI-RADS classification. Clinical records and imaging data were retrieved from the hospital’s Picture Archiving and Communication System (PACS).

### Participant and data collection

#### Participants

Adult patients aged 18 years or older with palpable breast masses were included. When multiple masses were present, the lesion with the most suspicious features was recorded. Patients were excluded if they had a prior histopathologic diagnosis before imaging or were classified as BI-RADS 0 (incomplete study) or BI-RADS 6 (known malignancy).

#### Classification framework

BI-RADS categories reflect an increasing probability of malignancy and provide standardized guidance for clinical management. The reference classification was based on mammography and/or breast ultrasound, with predicted classifications later detailed in the statistical analysis section (Table 1).

**Table 1:** Scheme of Reference and Predicted BI-RADS Classifications With Likelihood of Malignancy and Management, Modified From the ACR BI-RADS Fifth Edition (2013)

Reference classification (ACR BI-RADS fifth edition)	Likelihood of cancer	Management	Predicted classification	Chance of malignancy
BI-RADS 1 (negative); BI-RADS 2 (benign)	Essentially 0%	Routine screening	Predicted BI-RADS 1–2	Low
BI-RADS 3 (probably benign)	> 0% but ≤ 2%	Short-interval follow-up (6 months)	Predicted BI-RADS 3	Medium
BI-RADS 4 (suspicious)	> 2% to < 95%	Tissue diagnosis	Predicted BI-RADS 4–5	High
BI-RADS 5 (highly suggestive of malignancy)	≥ 95%			

ACR: American College of Radiology; BI-RADS: Breast Imaging Reporting and Data System.

#### Endpoints

The primary endpoint was the reference BI-RADS category (1–5), assigned according to the ACR BI-RADS fifth edition. For analysis, categories were grouped into three ordinal levels: BI-RADS 1–2 (low risk), BI-RADS 3 (medium risk), and BI-RADS 4–5 (high risk) (Table 1).

#### Candidate predictors

The ultrasound-retrieval team—comprising three independent radiologists (inter-observer kappa = 0.82)—was blinded to the final BI-RADS classification. Seven ultrasound features were evaluated as candidate predictors, following definitions in the ACR BI-RADS fifth edition (2013) [7].

##### 1) Shape (oval, round, irregular)

Oval shapes are typically benign, whereas irregular shapes strongly suggest malignancy.

##### 2) Margin (circumscribed, angular, microlobulated, indistinct, spiculated)

Benign masses are more often circumscribed, while malignant lesions frequently demonstrate the latter four patterns.

##### 3) Orientation (parallel vs. non-parallel)

Parallel orientation tends to be benign, whereas non-parallel suggests malignant invasion across tissue planes [11].

##### 4) Echo pattern (anechoic, hypoechoic, isoechoic, hyperechoic, heterogeneous, complex cystic-solid)

Anechoic or hyperechoic lesions are generally benign, whereas hypoechoic, heterogeneous, or complex cystic-solid patterns raise concern for malignancy.

##### 5) Posterior acoustic features (enhancement, shadowing, both, none)

Enhancement favors benignity, while shadowing suggests malignancy.

##### 6) Calcification (in a mass, outside a mass, intraductal)

Calcifications outside a mass are typically linked to non-mass findings and were not the focus of this study on palpable breast masses; in contrast, calcifications within a mass (especially punctate echogenic foci) and intraductal types are suspicious for malignancy.

##### 7) Axillary lymph node (normal, abnormal, not visualized)

Abnormal nodes, defined by cortical thickening, round shape, or hilum loss, are associated with malignancy [12].

#### Blinding and data transcription

To minimize information bias, ultrasound feature extraction was performed by the ultrasound-retrieval team, which was responsible solely for reviewing ultrasound images and retrieving ultrasound predictors. The researcher provided this team with ultrasound images that had all patient identifiers removed, including patient name, hospital number, and the final BI-RADS classification. The ultrasound-retrieval team had no access to any clinical information or outcome data.

Separately, the outcome team extracted the final BI-RADS classifications from clinical records independently, without access to the ultrasound predictors. Both teams worked entirely independently, and neither team had access to the other’s data during the process.

#### Specific time point for prediction

Predictions were generated immediately after completion of the breast ultrasound examination in patients with palpable breast masses. This timing reflects how the tool is intended to be used in practice—directly after ultrasound acquisition and before arranging the mammography queue or referral for confirmatory imaging.

### Study size estimation

Using the method for multivariable prediction models with a binary outcome described by Riley et al [13], and assuming a 50% prevalence of high-risk cases (BI-RADS 4–5), seven ultrasound parameters for predicting palpable breast masses, a C-statistic of 0.90, and a shrinkage factor of 0.90, the required sample size was 193 cases for high risk (BI-RADS 4–5) and 192 cases for low to medium risk (BI-RADS 1–3), yielding a total of 385 cases.

### Statistical analysis

#### Model derivation

The outcome variable comprised three categories: BI-RADS 1–2, BI-RADS 3, and BI-RADS 4–5. Although ordinal logistic regression would typically be used to develop such a model, some strata contained zero cases, leading to undefined odds ratios due to division by zero. To overcome this, all seven ultrasound features were entered into a two-step multivariable risk difference regression. Step 1 distinguished low-risk cases (BI-RADS 1–2) from those requiring further evaluation (BI-RADS 3–5). Step 2 then separated BI-RADS 3 (eligible for short-interval follow-up) from high-risk cases (BI-RADS 4–5) requiring expedited management. For each step, risk differences, 95% confidence intervals (CIs), and P values were reported.

#### Model performance and internal validation

For each step, discrimination was evaluated using the area under the receiver operating characteristic curve (AuROC) with 95% CI, estimated through either non-parametric or parametric methods. Calibration was evaluated by comparing predicted versus observed probabilities across risk strata and visually through calibration plots. Model optimism was quantified using bootstrap resampling with 200 cycles, and the bias-corrected performance was reported. Overall accuracy, as well as proportions of underestimation and overestimation, was summarized using confusion matrices.

#### Identifying cut points for clinical implications

Cut points were determined from risk curves generated by each model. In step 1, the threshold separating BI-RADS 3–5 from BI-RADS 1–2 was defined by the overall prevalence of BI-RADS 3–5 (y-axis). In step 2, the threshold distinguishing BI-RADS 4–5 from BI-RADS 3 was defined by the prevalence of BI-RADS 4–5 (y-axis) within the combined BI-RADS 3–5 group. In both steps, these prevalence values corresponded to points on the x-axis, which represented the linear combination from the models.

This study was registered in the Thai Clinical Trials Registry (TCTR) under the identifier TCTR20250826002. Ethical approval was obtained from the Institutional Review Board of Lampang Hospital (CERT No. 206.1/66). Owing to the observational nature of the research, the need for informed consent was waived. The study was conducted in accordance with the principles of the Declaration of Helsinki and adhered to the Transparent Reporting of a Multivariable Prediction Model for Individual Prognosis or Diagnosis (TRIPOD) guidelines [14] for its conduct and reporting.

## Results

### Study population

**Figure 1:**
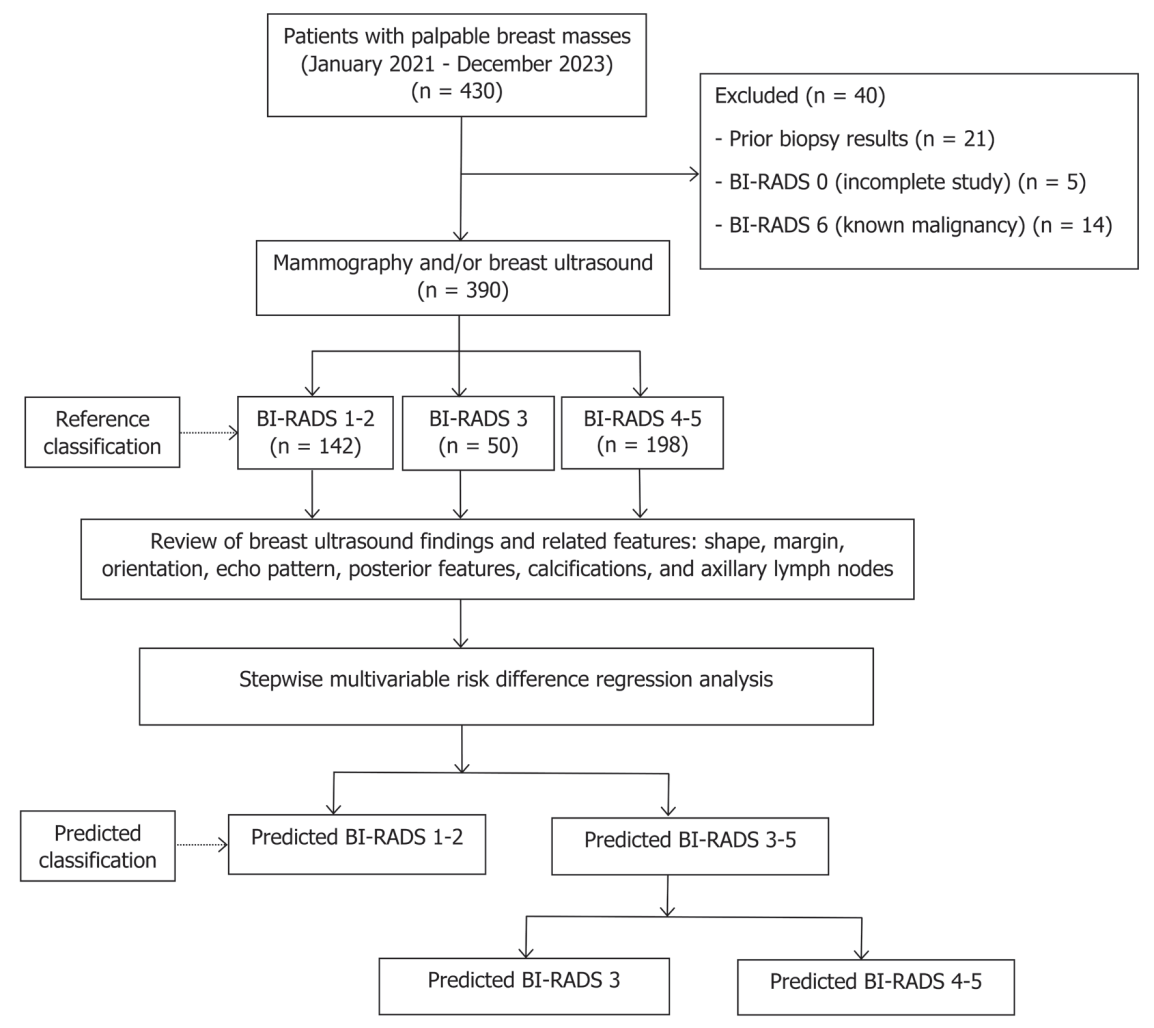
Study flow diagram. BI-RADS: Breast Imaging Reporting and Data System.

After exclusions, 390 patients were eligible. The reference BI-RADS classification comprised BI-RADS 1–2 (n = 142), BI-RADS 3 (n = 50), and BI-RADS 4–5 (n = 198). All seven ultrasound parameters were retrospectively reviewed. Predicted classifications were obtained from a two-step multivariable risk difference regression model: step 1 distinguished BI-RADS 3–5 from BI-RADS 1–2, and step 2 further separated BI-RADS 4–5 from BI-RADS 3 (Fig. 1).

### Baseline characteristics

Patients with BI-RADS 4–5 were the oldest group. Ultrasound findings associated with BI-RADS 4–5 included irregular shape, non-circumscribed margins, non-parallel orientation, heterogeneous or complex cystic-solid echogenicity, posterior acoustic shadowing, presence of calcifications, and abnormal axillary nodes (Table 2).

**Table 2:** Baseline Characteristics and Ultrasound Findings Across BI-RADS Categories

Variables	BI-RADS 1–2 (n = 142), n (%)	BI-RADS 3 (n = 50), n (%)	BI-RADS 4–5 (n = 198), n (%)	P value
Age (years), mean ± SD	49.7 ± 9.6	50.4 ± 11.1	58.7 ± 12.8	0.001
Ultrasound findings				
Shape				
None	28 (19.7)	0 (0)	0 (0)	< 0.001
Oval	111 (78.2)	47 (94.0)	46 (23.2)	
Round	1 (0.7)	2 (4.0)	9 (4.5)	
Irregular	2 (1.4)	1 (2.0)	143 (72.2)	
Margin				
None	28 (19.7)	0 (0)	0 (0)	< 0.001
Circumscribed	114 (80.3)	48 (96.0)	40 (20.2)	
Angular	0 (0)	0 (0)	19 (9.6)	
Microlobulated	0 (0)	0 (0)	27 (14.1)	
Indistinct	0 (0)	2 (4.0)	62 (31.3)	
Spiculated	0 (0)	0 (0)	50 (25.3)	
Orientation				
None	28 (19.7)	0 (0)	0 (0)	< 0.001
Parallel	114 (80.1)	49 (98.0)	120 (60.6)	
Not parallel	0 (0)	1 (2.0)	78 (39.4)	
Echo pattern				
None	28 (19.7)	0 (0)	0 (0)	< 0.001
Anechoic	104 (73.2)	8 (16.0)	0 (0)	
Hyperechoic	0 (0)	2 (4.0)	0 (0)	
Hypoechoic	10 (7.0)	37 (74.0)	114 (57.6)	
Isoechoic	0 (0)	2 (4.0)	1 (0.5)	
Heterogeneous	0 (0)	1 (2.0)	67 (33.8)	
Complex cystic-solid	0 (0)	0 (0)	16 (8.1)	
Posterior features				
None	28 (19.7)	0 (0)	0 (0)	< 0.001
No posterior features	28 (19.7)	43 (86.0)	94 (47.5)	
Enhancement	86 (61.6)	7 (14.0)	78 (49.4)	
Shadowing	0 (0)	0 (0)	25 (12.6)	
Combined pattern	0 (0)	0 (0)	1 (0.5)	
Calcifications				
None	28 (19.7)	0 (0)	0 (0)	< 0.001
No calcifications	114 (80.3)	50 (100)	117 (59.1)	
Calcifications in a mass	0 (0)	0 (0)	80 (40.4)	
Intraductal calcifications	0 (0)	0 (0)	1 (0.5)	
Axillary lymph nodes				
None	35 (13.0)	0 (0)	0 (0)	< 0.001
Normal	106 (36.7)	48 (16.6)	135 (46.7)	
Abnormal	0 (0)	0 (0)	57 (28.7)	

BI-RADS: Breast Imaging Reporting and Data System; SD: standard deviation; None: no mass.

### Significant predictors

After applying stepwise multivariable risk difference regression analysis, the original seven ultrasound predictors were reduced to three key variables—shape, margin, and echo pattern.

In step 1, oval, round, and irregular shapes contributed positive values to the linear combination for BI-RADS 3–5. Clinically, only irregular shape represented a meaningful increased risk. Circumscribed margin showed a trend toward decreased risk, and anechoic echogenicity was associated with decreased risk (Table 3).

**Table 3:** Step 1: Multivariable Risk Difference Regression With the stepwise Method of Ultrasound Predictors for Predicted BI-RADS 3–5 Versus BI-RADS 1–2 (Baseline)

Predictors	Risk difference	95% CI	P value
Shape			
None	Baseline	Baseline	Baseline
Oval	1.06	0.89, 1.24	< 0.001
Round	1.30	1.05, 1.54	< 0.001
Irregular	1.08	0.94, 1.23	< 0.001
Margin			
None	Baseline	Baseline	Baseline
Circumscribed	−0.11	−0.22, 0.01	0.069
Angular	−0.00	−0.11, 0.11	0.980
Microlobulated	−0.01	−0.11, 0.09	0.840
Indistinct	−0.01	−0.09, 0.07	0.801
Echo pattern			
None	Baseline	Baseline	Baseline
Anechoic	−0.89	−1.00, −0.78	< 0.001
Hyperechoic	−0.07	−0.39, 0.24	0.651
Hypoechoic	−0.10	−0.20, 0.01	0.081
Isoechoic	−0.03	−0.28, 0.23	0.833
Heterogeneous	−0.06	−0.17, 0.06	0.329

BI-RADS: Breast Imaging Reporting and Data System; CI: confidence interval; None: no mass.

In step 2, the same three predictors remained as the final variables: round and irregular shapes, non-circumscribed margins (angular, microlobulated, indistinct, spiculated), plus hypoechoic, heterogeneous, or complex cystic and solid echogenicity, all of which were significantly associated with increased risk of BI-RADS 4–5 (Table 4). Clinically, round shape may suggest malignancy in about half of cases, whereas irregular shape is more consistently associated with malignant potential.

**Table 4:** Step 2: Multivariable Risk Difference Regression Using the Stepwise Method of Ultrasound Predictors for Predicted BI-RADS 4–5 Versus BI-RADS 3 (Baseline)

Predictors	Risk difference	95% CI	P value
Shape			
None	Baseline	Baseline	Baseline
Round	0.50	0.21, 0.79	0.001
Irregular	0.15	0.01, 0.28	0.030
Margin			
None	Baseline	Baseline	Baseline
Angular	0.40	0.22, 0.57	< 0.001
Microlobulated	0.36	0.19, 0.53	< 0.001
Indistinct	0.35	0.21, 0.49	< 0.001
Spiculated	0.39	0.23, 0.55	< 0.001
Echo pattern			
None	Baseline	Baseline	Baseline
Hypoechoic	0.67	0.26, 1.07	0.001
Isoechoic	0.30	−0.21, 0.81	0.249
Heterogeneous	0.81	0.40, 1.22	< 0.001
Complex cystic-solid	1.06	0.64, 1.49	< 0.001

BI-RADS: Breast Imaging Reporting and Data System; CI: confidence interval; None: no mass.

### Model specification

The final two-step model equation is provided below to allow calculation of predicted probabilities for individual patients.

#### Step 1

Predict prob = 0 + 1.063063 × *x*_1_ + 1.296871 × *x*_2_ + 1.083918 × *x*_3_ - 0.1073534 × *x*_4_ - 0.0013926 × *x*_5_ - 0.0100573 × *x*_6_ - 0.0101208 × *x*_7_ - 0.8888282 × *x*_8_ - 0.0726132 × *x*_9_ - 0.0956113 × *x*_10_ - 0.0274826 × *x*_11_ - 0.057393 × *x*_12_

where *x*_1_ is oval shape, *x*_2_ is round shape, *x*_3_ is irregular shape, *x*_4_ is circumscribed margin, *x*_5_ is angular margin, *x*_6_ is microlobulated margin, *x*_7_ is indistinct margin, *x*_8_ is anechoic, *x*_9_ is hyperechoic, *x*_10_ is hypoechoic, *x*_11_ is isoechoic, and *x*_12_ is heterogeneous echogenicity.

#### Step 2

Predict prob = -0.2507609 + 0.5015217 × *x*_1_ + 0.1489587 × *x*_2_ + 0.3952947 × *x*_3_ + 0.3599356 × *x*_4_ + 0.3503577 × *x*_5_ + 0.3926078 × *x*_6_ + 0.6656522 × *x*_7_ + 0.3008695 × *x*_8_ + 0.8082629 × *x*_9_ + 1.06232 × *x*_10_

where *x*_1_ is round shape, *x*_2_ is irregular shape, *x*_3_ is angular margin, *x*_4_ is microlobulated margin, *x*_5_ is indistinct margin, *x*_6_ is spiculated margin, *x*_7_ is hypoechoic, *x*_8_ is isoechoic, *x*_9_ is heterogeneous echogenicity, and *x*_10_ is complex cystic-solid echogenicity.

### Model performances

#### Discrimination

**Figure 2:**
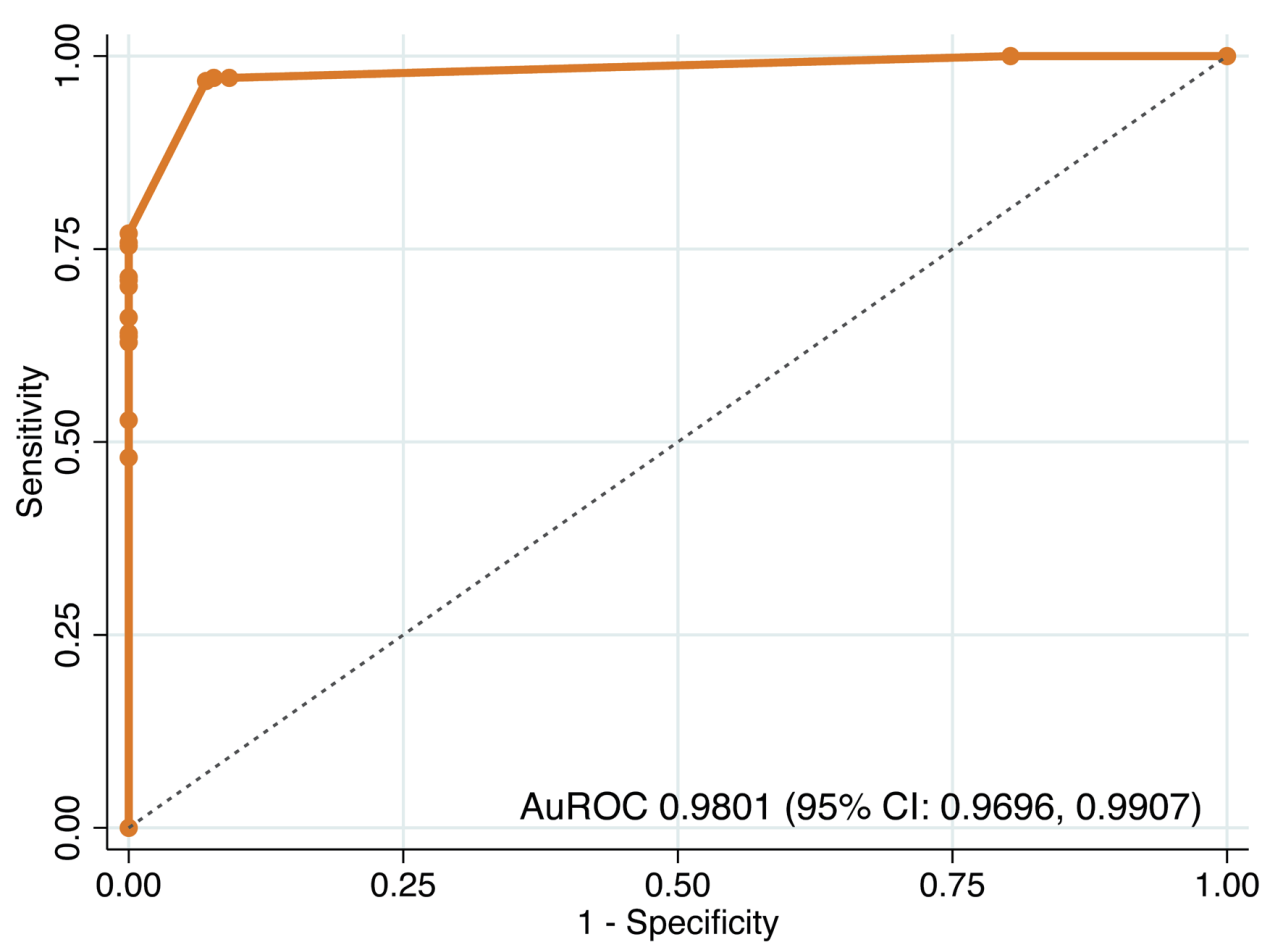
Receiver operating characteristic (ROC) curve of step 1 model for differentiating BI-RADS 3–5 from BI-RADS 1–2. AuROC: area under the receiver operating characteristic curve; CI: confidence interval.

**Figure 3:**
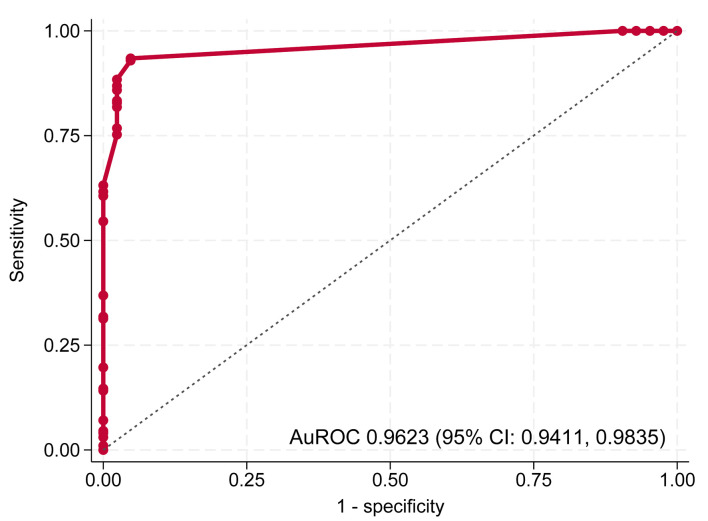
Receiver operating characteristic (ROC) curve of step 2 model for differentiating BI-RADS 4–5 from BI-RADS 3. AuROC: area under the receiver operating characteristic curve; CI: confidence interval.

The predictive model demonstrated excellent discriminative ability in both steps. In step 1, distinguishing BI-RADS 3–5 from BI-RADS 1–2, the model achieved an AuROC of 0.9801 (95% CI, 0.9696–0.9907) (Fig. 2). In step 2, distinguishing BI-RADS 4–5 from BI-RADS 3, the model maintained high performance with an AuROC of 0.9623 (95% CI, 0.9411–0.9835) (Fig. 3).

#### Calibration

Internal validation with 200 bootstrap cycles showed minimal optimism in both steps. In step 1, discrimination and calibration were excellent (AuROC 0.9800, slope 0.9800, E:O ratio 1.0010, calibration-in-the-large (CITL) −0.0240). In step 2, discrimination remained strong (AuROC 0.9630), though the calibration slope indicated slight overfitting (0.8960). Overall, bootstrap shrinkage values (0.9800 and 0.8960) confirmed good internal validity of the models (Table 5).

**Table 5:** Apparent and Bootstrap Performance of Step 1 and Step 2 Prediction Models

Process	Parameters	Apparent performance	Bootstrap performance
Step 1	AuROC	0.9801 (0.9696, 0.9907)	0.9800 (0.9710, 0.9910)
	Slope	1.0000 (0.8390, 1.1610)	0.9800 (0.8230, 1.1450)
	E:O ratio	1.0000	1.0010 (0.9720, 1.0410)
	CITL	−0.0000 (−0.4850, 0.4850)	−0.0240 (−0.6580, 0.5700)
	Bootstrap shrinkage	NA	0.9800
Step 2	AuROC	0.9623 (0.9411, 0.9835)	0.9630 (0.9420, 0.9900)
	Slope	1.0000 (0.7110, 1.2890)	0.8960 (0.0000, 1.2830)
	E:O ratio	1.0000	0.9190 (0.2500, 1.0240)
	CITL	0.0000 (−0.5810, 0.5810)	0.0700 (−0.4270, 0.8750)
	Bootstrap shrinkage	NA	0.8960

AuROC: area under the receiver operating characteristic curve; CITL: calibration-in-the-large; E:O ratio: expected-to-observed outcomes ratio; NA: not applicable.

#### Cut point threshold selection

**Figure 4:**
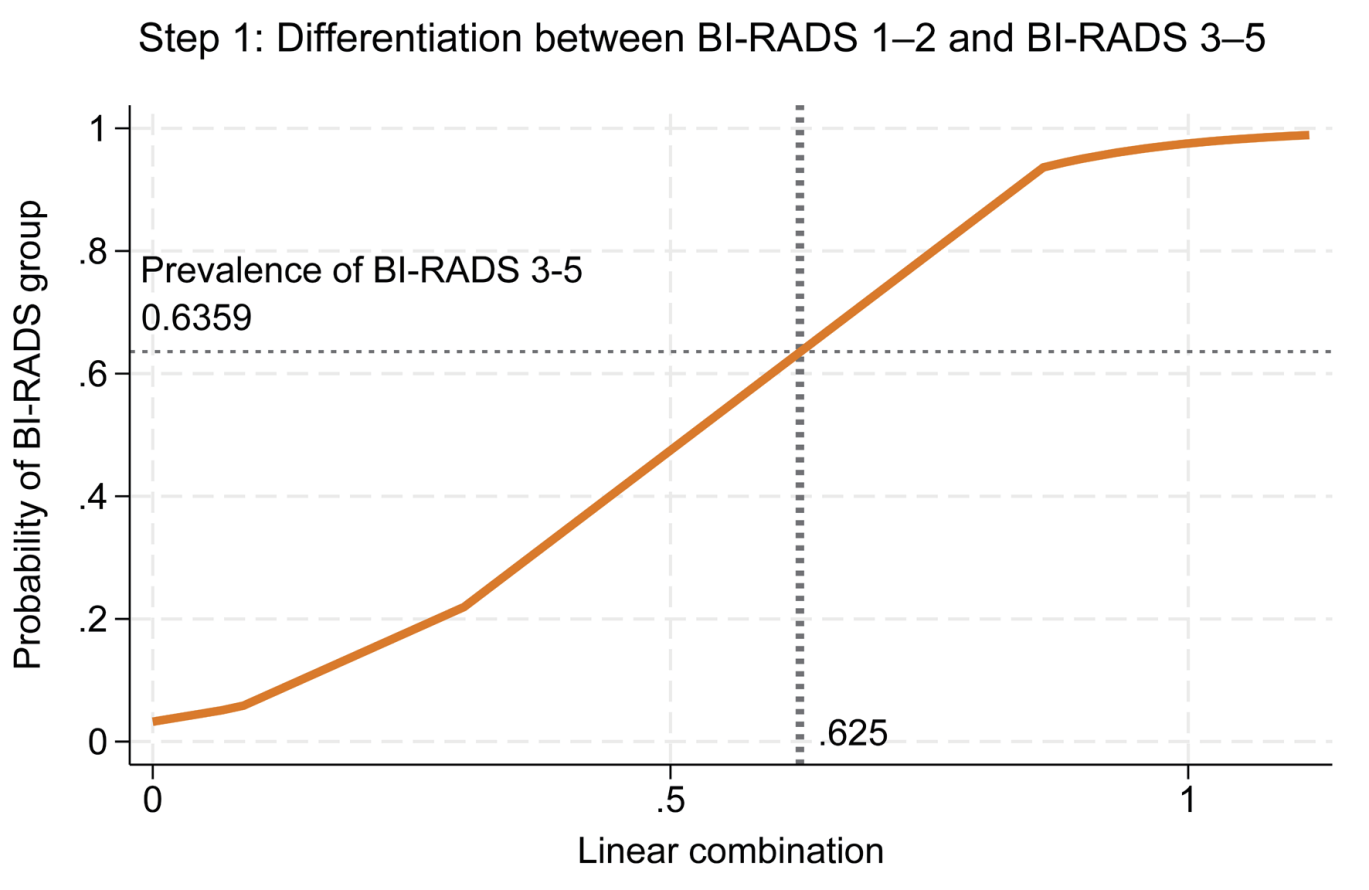
Risk curve showing the probability of BI-RADS 3–5 with BI-RADS 1–2 as baseline and the cutoff point of the linear combination. BI-RADS: Breast Imaging Reporting and Data System.

**Figure 5:**
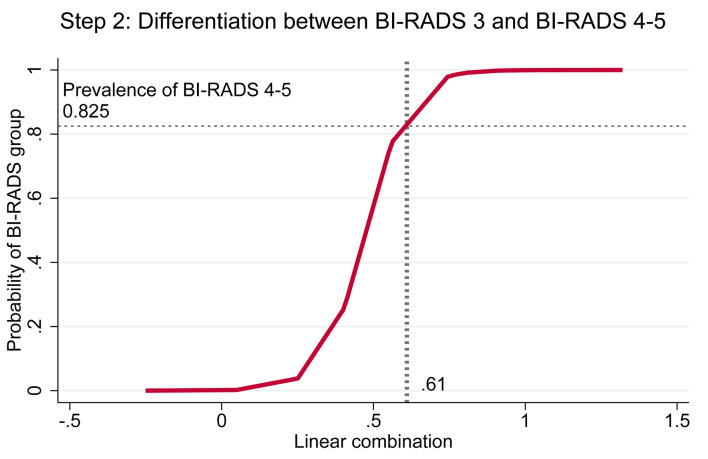
Risk curve showing the probability of BI-RADS 4–5 with BI-RADS 3 as baseline and the cutoff point of the linear combination. BI-RADS: Breast Imaging Reporting and Data System.

Cutoff thresholds were determined using risk curves for both steps. In step 1, the prevalence of BI-RADS 3–5 among BI-RADS 1–5 was 0.6359, which intersected with the linear combination (*xb*) at 0.625; values beyond this point indicated classification as BI-RADS 3–5 (Fig. 4). In step 2, the prevalence of BI-RADS 4–5 among BI-RADS 3–5 was 0.825, intersecting with *xb* at 0.61; values above this threshold indicated classification as BI-RADS 4–5 (Fig. 5).

#### Confusion matrix

Percentages in each cell were calculated as n/390. The overall accuracy, based on diagonal cells, was 88.5%. Overprediction occurred in 2.5% (predicted BI-RADS 3 for reference BI-RADS 1–2), 0% (predicted BI-RADS 4–5 for reference BI-RADS 1–2), and 0.3% (predicted BI-RADS 4–5 for reference BI-RADS 3), with no instances exceeding one step. Underprediction was observed in 2.0% (predicted BI-RADS 1–2 for reference BI-RADS 3) and 6.7% (predicted BI-RADS 3 for reference BI-RADS 4–5), but none exceeded one step (Table 6).

**Table 6:** Confusion Matrix of Predicted Versus Reference BI-RADS Classifications

Predicted classification	Reference classification			Total
	BI-RADS 1–2 (n = 142)	BI-RADS 3 (n = 50)	BI-RADS 4–5 (n = 198)	
Predicted BI-RADS 1–2	132 (33.9%)	8 (2.0%)	0 (0%)	140 (35.9%)
Predicted BI-RADS 3	10 (2.5%)	41 (10.5%)	26 (6.7%)	77 (19.7%)
Predicted BI-RADS 4–5	0 (0%)	1 (0.3%)	172 (44.1%)	173 (44.4%)
Total	142 (36.4%)	50 (12.8%)	198 (50.8%)	390 (100%)

BI-RADS: Breast Imaging Reporting and Data System.

## Discussion

Palpable breast masses typically require subsequent mammography. In Thailand, where hospital access is relatively easy, this often results in overcrowded services. Without re-arranging the current queue, a substantial proportion of patients face delayed diagnosis and BI-RADS classification, which in turn postpones timely treatment. Conversely, if almost all patients are prioritized as urgent, the workload for technicians and radiologists becomes overwhelming, and truly urgent cases may experience delays in diagnosis and classification.

A small number of discordant cases were identified in Table 2, where an “irregular shape” was present in lesions ultimately categorized as benign BI-RADS levels. These discrepancies can be explained by two clinically plausible scenarios. First, for one BI-RADS 3 lesion, the routine radiology report described a “lobulated mass” without specifying the shape; however, structured re-evaluation by the ultrasound-retrieval team identified an irregular shape accompanying the lobulated margin. Second, two BI-RADS 2 lesions with irregular shape corresponded to postoperative scars from remote prior biopsies, unrelated to the current episode, where benign architectural distortion can mimic an irregular contour. These uncommon findings reflect real-world variability between routine narrative reporting and structured ultrasound feature extraction.

Shape, margin, and echo pattern demonstrated strong predictive performance in both step 1 and step 2 of BI-RADS classification in this study. This finding is consistent with a previous report showing that these three features were significant predictors in tumors ≤ 1 cm [15]. In contrast, other studies have noted that irregular margins and marked hypoechogenicity remained predictive of malignancy irrespective of lesion size [16]. Taken together, these three ultrasound characteristics provide valuable information for predicting BI-RADS classification.

Most previous studies have applied risk regression or logistic regression for binary outcomes. In our study, the outcome comprised three categories—BI-RADS 1–2, 3, and 4–5—representing increasing severity from low to high, making it an ordinal outcome. The appropriate approach would have been an ordinal logistic regression model; however, the presence of zero values in some cells made this method inapplicable. To address this issue, we employed a two-step model using risk difference regression analysis.

The model demonstrated excellent performance. In step 1, distinguishing BI-RADS 3–5 from BI-RADS 1–2, the AuROC was 0.9801 (95% CI, 0.9696–0.9907). In step 2, distinguishing BI-RADS 4–5 from BI-RADS 3, the AuROC remained high at 0.9623 (95% CI, 0.9411–0.9835). Internal validation with 200 bootstrap cycles showed minimal optimism in both steps, with calibration slopes close to unity and bootstrap shrinkage values of 0.9800 and 0.8960, confirming good internal validity of the models.

Nearly all patients (99.5%) underwent both mammography and breast ultrasound, in accordance with ACR guidelines [7]. Dense breasts limit the ability of mammography to detect abnormalities, whereas additional ultrasound can improve sensitivity by providing greater detail and better characterization of masses [17]. Among the 390 patients, interpretation relied primarily on mammography in 172 (44.1%), while 218 (55.9%) required ultrasound as the reference modality. This raised concerns regarding incorporation bias. To address this issue, sensitivity analysis was performed by comparing model performance between the overall cohort and mammography-based interpretation alone. Discrimination remained excellent in both steps: for step 1, AuROC was 0.9801 (95% CI, 0.9695–0.9907) and 0.9945 (95% CI, 0.9866–1.0000), and for step 2, 0.9623 (95% CI, 0.9411–0.9835) and 0.9876 (95% CI, 0.9707–1.0000), respectively. These findings indicate that incorporation bias, if present, had minimal impact on diagnostic performance, supporting the robustness of the model (Table 7).

**Table 7:** Sensitivity Analysis of Discriminative Ability by Method (Addressing Incorporation Bias)

Process	Method	Patients (n)	AuROC	95% CI
Step 1	Overall model	390	0.9801	0.9695, 0.9907
	Mammography-based interpretation alone	172	0.9945	0.9866, 1.0000
Step 2	Overall model	240	0.9623	0.9411, 0.9835
	Mammography-based interpretation alone	163	0.9876	0.9707, 1.0000

AuROC: area under the receiver operating characteristic curve; CI: confidence interval.

Among BI-RADS 4–5 lesions (n = 198), tissue diagnosis was obtained in 195 cases, of which 157 were malignant (80.5%) and 38 were benign (19.5%). The most common malignancy was invasive ductal carcinoma, accounting for 81.6% of cancers, which is consistent with previous reports of approximately 86.6% [18]. Smaller proportions consisted of ductal carcinoma *in situ* and other less common subtypes. This distribution aligns with the typical epidemiologic pattern of breast malignancies and supports the validity of the reference BI-RADS assignments.

Misclassification was identified in this study, with underestimation representing the main concern. In particular, 6.7% of cases were predicted as BI-RADS 3 while the reference standard was BI-RADS 4–5, raising potential concern about delayed detection of disease progression. To mitigate this, a modified follow-up schedule was proposed: patients predicted as BI-RADS 3 would be reassigned to a shorter mammography queue of 4–8 weeks, compared with within 4 weeks for BI-RADS 4–5. The 4-week difference between these categories is unlikely to permit meaningful disease progression (Table 8). By contrast, overestimation occurred in fewer than 3% of cases, which could help reduce the mammography backlog and lessen the overall clinical workload.

**Table 8:** Model-Based Scheduling Scheme: A New Strategy for Mammography Queue Management

Process	Linear combination	Predicted group	Chance of malignancy	Queue	Waiting time
Step 1	< 0.625	BI-RADS 1–2	Low	Normal	Standard queue
	≥ 0.625	BI-RADS 3–5	Proceed to step 2 evaluation		
Step 2	< 0.610	BI-RADS 3	Medium	Semi-urgent	Within 4 - 8 weeks
	≥ 0.610	BI-RADS 4–5	High	Urgent	Within 4 weeks

BI-RADS: Breast Imaging Reporting and Data System.

This study has several limitations. First, its retrospective, single-center design may limit generalizability, as the model was derived from patients at a tertiary referral hospital with a relatively high prevalence of BI-RADS 4–5 lesions. Second, although nearly all patients underwent both mammography and ultrasound, the interpretation’s reliance on ultrasound as the reference modality in women with dense breasts may have introduced incorporation bias. While sensitivity analyses indicated minimal impact, residual bias could not be entirely eliminated. Third, misclassification was inevitable: underestimation occurred in 6.7% of cases. To address this, a rescheduling strategy was proposed, in which BI-RADS 3 cases would be reassigned to 4–8 weeks and BI-RADS 4–5 to within 4 weeks. Reassessment after implementation of this model in real-world practice will be necessary. Moreover, external validation was not performed. Although internal performance was strong, prediction models often show reduced accuracy when applied to new populations with different patient characteristics, imaging equipment, and operator variability. Validation in independent cohorts across other institutions is planned as the next phase to confirm generalizability before wider clinical adoption.

To facilitate practical application, this two-step model has been implemented as an online tool [19].

### Conclusions

A two-step breast ultrasound model incorporating shape, margin, and echo pattern demonstrated strong discrimination and calibration with minimal optimism. The model achieved high accuracy, with slightly elevated underestimation but minimal overestimation. This re-scheduling strategy optimizes mammography queue prioritization, reduce delays, and balance radiology workloads. External validation is necessary before clinical implementation.

## Data Availability

Any inquiries regarding supporting data availability of this study should be directed to the corresponding author.

## References

[1] https://www.who.int/news-room/fact-sheets/detail/breast-cancer.

[2] https://www.wcrf.org/preventing-cancer/cancer-statistics/breast-cancer-statistics.

[3] Maroni R, Massat NJ, Parmar D, Dibden A, Cuzick J, Sasieni PD, Duffy SW (2021). A case-control study to evaluate the impact of the breast screening programme on mortality in England. Br J Cancer.

[4] Tabar L, Vitak B, Chen TH, Yen AM, Cohen A, Tot T, Chiu SY (2011). Swedish two-county trial: impact of mammographic screening on breast cancer mortality during 3 decades. Radiology.

[5] Berg WA, Blume JD, Cormack JB, Mendelson EB, Lehrer D, Bohm-Velez M, Pisano ED (2008). Combined screening with ultrasound and mammography vs mammography alone in women at elevated risk of breast cancer. JAMA.

[6] Parmar J, Choudhary S, Zope A, Patel T, Chaudhari N, Shah S (2022). Comprehensive comparison of diagnostic accuracy of ultrasound and mammography in young women with radiographically dense breasts. Arch Clin Biomed Res.

[7] D’Orsi CJ, Sickles EA, Mendelson EB, Morris EA, American College of Radiology (2013). ACR BI-RADS atlas: breast imaging reporting and data system; mammography, ultrasound, magnetic resonance imaging, follow-up and outcome monitoring, data dictionary. 5th ed.

[8] Gharekhanloo F, Haseli MM, Torabian S (2018). Value of ultrasound in the detection of benign and malignant breast diseases: a diagnostic accuracy study. Oman Med J.

[9] Kumar BV, Kumar AR (2018). Ultrasound evaluation of breast masses and histopathology correlation. Int J Contemp Med Sur Radio.

[10] Akthar H, Patil NA (2023). Combined mammography and ultrasound evaluation of palpable breast masses with pathological correlation. Indian J Basic Appl Med Res.

[11] Chen K, Wu S (2024). The utility of quantifying the orientation of breast masses in ultrasound imaging. Sci Rep.

[12] Choi YJ, Ko EY, Han BK, Shin JH, Kang SS, Hahn SY (2009). High-resolution ultrasonographic features of axillary lymph node metastasis in patients with breast cancer. Breast.

[13] Riley RD, Snell KI, Ensor J, Burke DL, Harrell FE, Moons KG, Collins GS (2019). Minimum sample size for developing a multivariable prediction model: PART II - binary and time-to-event outcomes. Stat Med.

[14] Collins GS, Reitsma JB, Altman DG, Moons KG (2015). Transparent reporting of a multivariable prediction model for individual prognosis or diagnosis (TRIPOD): the TRIPOD statement. BMJ.

[15] Chen SC, Cheung YC, Su CH, Chen MF, Hwang TL, Hsueh S (2004). Analysis of sonographic features for the differentiation of benign and malignant breast tumors of different sizes. Ultrasound Obstet Gynecol.

[16] Del Frate C, Bestagno A, Cerniato R, Soldano F, Isola M, Puglisi F, Bazzocchi M (2006). Sonographic criteria for differentiation of benign and malignant solid breast lesions: size is of value. Radiol Med.

[17] Melnikow J, Fenton JJ, Whitlock EP, Miglioretti DL, Weyrich MS, Thompson JH, Shah K (2016). Supplemental screening for breast cancer in women with dense breasts: a systematic review for the U.S. preventive services task force. Ann Intern Med.

[18] Sirikunakorn P, Marukatat N, Tangjitgamol S, Loharamtaweethong K (2014). Positive predictive value of malignancy in BI-RADS 4 and 5 breast lesions. Vajira Med J.

[19] https://tharathipdevelop.com/birads2/form/with-img/2.

